# Assessing Age-Related Gray Matter Differences in Young Adults with Voxel-Based Morphometry: The Effect of Field Strengths

**DOI:** 10.3390/brainsci11040447

**Published:** 2021-03-31

**Authors:** Feng-Yi Su, Jyun-Ru Chen, Chun-Ming Chen, Yen-Chih Huang, Shin-Lei Peng

**Affiliations:** 1 Department of Medical Imaging, China Medical University Hospital, Taichung 404333, Taiwan; t16832@mail.cmuh.org.tw (F.-Y.S.); jinmingc@yahoo.com.hk (C.-M.C.); arvin32.huang@gmail.com (Y.-C.H.); 2 Department of Biomedical Imaging and Radiological Science, China Medical University, Taichung 404333, Taiwan; r1c15j@gmail.com

**Keywords:** voxel-based morphometry (VBM), gray matter, field strength, visual cortex, cerebellum

## Abstract

Knowing the patterns of brain differences with age in the young population could lead to a better understanding of the causes of certain psychiatric disorders; however, relevant information is insufficient. Here, a pattern of regional gray matter (GM) that changed with age in a young cohort aged 20–30 years was provided. Extending from previous age studies, all participants were imaged at both 1.5 T and 3 T to address the question of how far the field strength influences results. Fifty-nine young participants aged 20–30 years were scanned at both 1.5 T and 3 T. Voxel-based morphometry (VBM) was used to estimate the GM volume. Some brain regions showed a significant field strength-dependent difference in GM volume. VBM uncovered a significantly age-related increase in the GM volume in the left visual-associated area at 3 T, which was not detected at 1.5 T. In addition, voxels at 1.5 T that revealed a significant age-related reduction in the GM volume were found in the right cerebellum. In conclusion, age-related differences in human brain morphology could even be detected in a young cohort aged 20–30 years; however, the results varied across field strengths. Thus, field strength should be considered an important factor when comparing age-specific brain differences across studies.

## 1. Introduction

Extensive literature has established that the human brain undergoes continuous structural changes with age, even in healthy adults [1,2,3]. By recruiting participants with a wider age range from adolescence, early adulthood, and middle adulthood to elderly age, one of the well-characterized findings is that global and regional gray matter (GM) volume decreases with age. The neurobiological basis of this structural shrinkage contributes to the following cognitive function decline and, therefore, neurodegenerative diseases [4]. However, whether there exists any pattern of age-related changes in young adults aged in the mid-to-late 20 s is unclear. Awareness is increasing that some psychiatric disorders showed a significant decreasing trend with older age, with a maximum incidence rate in the age group of the 20 s [5,6]. Knowledge of brain difference patterns with age in the young population could lead to a better understanding of the causes of certain psychiatric disorders, and perhaps offer benefits of preventive interventions. Despite its significance, the relative paucity of neuroimaging studies reporting age-associated effects on brain morphology specific to young adults as the pattern of GM volume changed with age can be relatively difficult to detect in young participants compared with elderly participants [7].

Complemented by a fully automated whole-brain analysis method, the so-called voxel-based morphometry (VBM), neuroanatomical studies using magnetic resonance imaging (MRI), have provided useful information with respect to the effect of age on brain morphometry [1,2,3,7]. Results across studies were only partly congruent, although VBM is a method of choice for investigating age-related effects on the brain. One explanation could be because as the series of preprocessing steps may complicate data interpretations, small methodological variations can have a major influence on VBM results. Age-specific differences in GM estimates are more pronounced using standard Statistical Parametric Mapping (SPM) VBM than those using Diffeomorphic Anatomical Registration Using Exponentiated Lie Algebra (DARTEL) VBM [1]. Moreover, comparisons of MRI-derived morphometric quantifications can be influenced not only by the aforementioned methodology-related factors but also by instrument-related factors, such as field strength [8,9]. Age-related changes in brain structures have been investigated using MRI principally at a 1.5 T field strength [2,7,10] but less commonly at 3 T [1] over the last two decades. Relaxation times depend on the main magnetic field strength; hence, the GM and white matter (WM) ratio of T1 relaxation times is higher at 1.5 T than at 3 T [11]. Therefore, expecting that signal intensity and image contrast vary across image platforms and subsequently affect VBM results is reasonable [8,9]. The question as to whether brain volume assessments across field strengths affect age-related differences throughout the brain is intriguing and warrants further investigation; however, no study to date has filled this gap.

As a significant clinical relevance of evaluating volumetric-brain differences in early adulthood was noted, we examined the normal brain and provided a pattern of regional GM differences with age in a young cohort aged 20–30 years. Extending from the previous age studies, all participants were imaged at both 1.5 T and 3 T to address the question of how far the field strength influences VBM results in terms of age-specific brain differences in a young brain. The findings of this study may help future work better understand idiosyncratic patterns of age-related differences in a young brain. 

## 2. Materials and Methods

### 2.1. Subjects and Study Design

In this study, the participants included 33 men (mean age, 23.8 ± 2.6 years; range = 20–30 years) and 26 females (mean age = 23 ± 2.27 years; range = 20–30 years). Handedness was determined using self-reports of hand preference. Only one female participant was left-handed. According to self-completed questionnaires, all participants were healthy and had no history of cardiovascular, psychiatric, or neurological diseases. No participant was a tobacco smoker, an alcohol drinker, and a drug user. All participants provided informed written consent after the study protocol was clearly explained to them. All participants underwent both 1.5 T and 3 T MRI scanning on the same day, and the order of scanning was randomized among participants. The time interval between the 1.5 T and 3 T MRI scans was at least 30 min. The local institutional review board approved the study protocol. 

### 2.2. MRI Protocol

All experiments were performed on a 1.5 T MR system (GE Optima MR450w, Milwaukee WI, USA) and a 3 T MR system (GE Signa HDxt, Milwaukee WI, USA) using a 16-channel coil. The participants were all fitted with foam pads to reduce head motion. For the 1.5 T scanner, axial T1-weighted (T1W) images were obtained using fast-spoiled gradient echo (FSPGR), repetition time (TR)/echo time (TE)/flip angle (FA) = 6.22 ms/1.99 ms/12°, time of inversion (TI) = 450 ms, spatial resolution = 1 × 1 × 1 mm^3^, and number of slices = 170. For the 3 T scanner, axial T1W images were acquired using FSPGR as well, TR/TE/FA = 8.02 ms/2.99 ms/12°, TI = 450 ms, spatial resolution = 1 × 1 × 1 mm^3^, and number of slices = 170.

### 2.3. Data Analysis

For each subject, FSL software (FMRIB Software Library, Oxford University, Oxford, UK, version 6.0.1) was used to segment T1W images into GM, WM, and cerebrospinal fluid (CSF) in the subject’s space. Both Brain Extraction Tool for skull stripping and FMRIB’s Automated Segmentation Tool for brain segmentation were run using the default parameters. The sum of GM, WM, and CSF was used to calculate the total intracranial volume (TIV). Both the cerebellum and brainstem were included.

To perform VBM, all T1W images were processed using SPM12 (http://www.fil.ion.ucl.ac.uk/spm/, version 12) on MATLAB (The MathWorks, Natick, MA, USA). All T1W images were spatially normalized using the DARTEL algorithm [12], and then segmented into GM, WM, and CSF. The resulting GM segments were normalized to Montreal Neurological Institute space and smoothed using a Gaussian kernel of 8 mm full width at half maximum.

### 2.4. Statistical Analysis

For VBM results, voxel-by-voxel differences in the GM volume between 3 T and 1.5 T were assessed using a two-tailed paired *t*-test. To assess age-related structural brain differences, VBM results from both field strengths were analyzed separately using the following multiple regression analysis:

Volume = b_0_ × intercept + b_1_ × age + b_2_ × sex + b_3_ × handedness + b_4_ × TIV (1)

Here, b values are the coefficients of the variables. To further address the effects of an interaction between age and field strength on the GM volume, we conducted another analysis by including the B_0_ and age × B_0_ interaction term in the regression model. For all analyses, the voxel-level threshold was set to *p <* 0.001 (uncorrected) and clusters of more than 100 voxels.

## 3. Results

### 3.1. Voxel-Wise Differences in GM between Field Strengths

**Figure 1:**
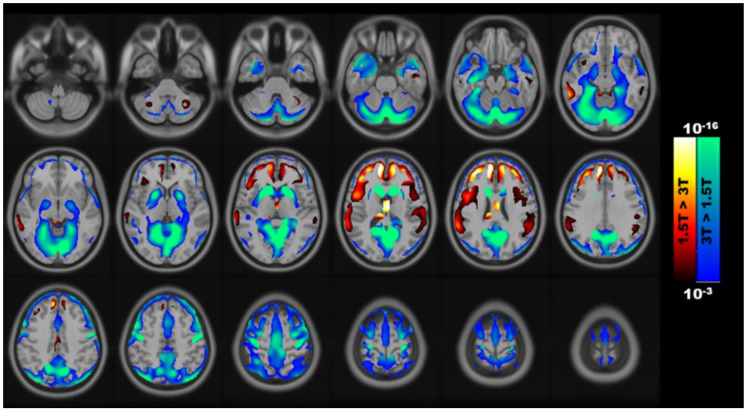
Voxels with field strength-dependent changes in the gray matter volume using VBM analysis.

A voxel-wise paired comparison of the relationship between the GM volume and field strength revealed that some brain regions had a significant field strength-dependent difference in GM volume (Figure 1). Positive clusters (1.5 T > 3 T) could be observed as largely distributed in the anterior regions such as the frontal cortex and corpus callosum. However, negative clusters (3 T > 1.5 T) are mainly located in the posterior regions such as the occipital and parietal lobes.

### 3.2. Age-Related Changes in the GM Volume between Field Strengths

**Figure 2:**
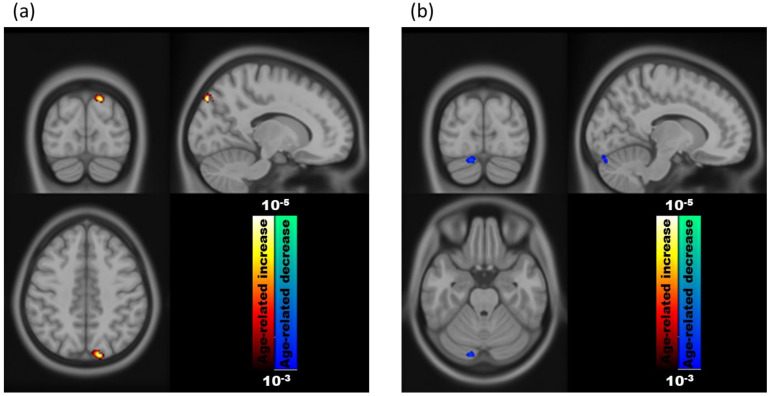
Age-related changes in the gray matter volume using VBM analysis at (**a**) 3 T and (**b**) 1.5 T.

VBM results of age-related differences in brain volumes are presented in Figure 2a,b for 3 T and 1.5 T, respectively. A significant field strength-dependent difference was observed in the VBM analysis. VBM revealed a significant age-related increase in the GM volume in the left visual-associated area (coordinate: −14 −85 30; size: 165; T_max_: 4.56) at 3 T, which was not detected at 1.5 T. In addition, voxels that revealed a significant age-related reduction in the GM volume at 1.5 T were found in the right cerebellum (coordinate: 15 −87 −35; size: 129; T_max_: 3.59). No brain regions demonstrated a significant age-related GM increase at 1.5 T. When including the age × B_0_ interaction term in the regression model, it had an insignificant effect on the GM volume.

## 4. Discussion

In this study, we showed that age-related differences in human brain morphology could be detected in a young cohort aged 20–30 years. Distinct from other studies, this study extended previous findings by showing that VBM results are significantly influenced by field strengths, as patterns of age-related differences are heterogeneous across field strengths. It is important to consider when comparing aging studies conducted at different field strengths for VBM analyses. 

As the existing literature has shown that the GM volume of an adult human brain significantly diminishes with age [1,2,13,14,15], one might argue that data acquired at 3 T with an age-related increase in the GM volume in visual-associated regions could occur as a potential overestimation at first glance. Theoretically, several significant challenges were found for imaging at 3 T compared with that at 1.5 T, such as magnetic field inhomogeneity [16]. Moreover, protruded parts such as the occipital lobes distant from the isocenter of the MRI scanners may suffer from increased magnetic field inhomogeneity. Technique consequences of field inhomogeneity and an off-center location may contribute to the inaccurate image contrast. However, in this study, both 3 T and 1.5 T systems were equipped with 16-channel coils. Coils with more receiving elements have shown to benefit B_0_ homogeneity improvement at 3 T [17]; thus, field inhomogeneity could be less considered in the design of this study. In addition, the intracortical myelin in the visual cortex continued to mature sequentially into adulthood and peak at approximately 34 years old [18], suggesting a prolonged development of the visual cortex [19]. With the compelling pieces of evidence from existing studies, the age-related increase in the GM volume in visual-associated regions observed in this study represents a faithful reflection of the effects of aging in a young cohort aged 20–30 years. 

The cerebellum is a brain region that is markedly enlarged in humans compared with that in other mammals [20]. Functional specificity of the cerebellum includes balance, motor control, and the ability to learn complex motor sequences. Moreover, the cerebellum plays a prominent role in cognitive and emotional functions [21,22]. Therefore, understanding the quantitative morphology of the cerebellum throughout the lifespan is a priority. A study by Tiemeier et al. has shown that the developmental trajectory of cerebellum volume peaked at early adolescence, at age 11.8 years and 15.6 years in women and men, respectively [23]. In this study, the age-related shrinkage of the cerebellum detected in a young population corresponds well with their finding of the onset of the cerebellum volume decrease after pubertal maturation. The cerebellum shrinkage has been implicated in several psychiatric disorders, such as schizophrenia [24] and anorexia [25]. As the symptoms of these psychiatric disorders start in early adulthood, cerebellum volume decreasing with age in a young cohort can be considered a prime target for these psychiatric neuroimaging investigations. 

VBM is as an interesting tool to quantify structural changes in the brain, and a series of studies have used VBM as a method of choice to search broadly for brain regions showing age-related differences [1,2]. As VBM depends heavily on the GM/WM contrast in MRI images, different scanning platforms can result in intensity and geometric variations. This contributes to the particular relevance in data analysis. Considering that each system has specific image contrasts and sources of error, the effect of field strength on the VBM analysis is a topic of interest [8,9]. Here, GM quantifications from the VBM analysis were demonstrated to be heterogeneous across different field strengths. Even though the tested sample size is relatively small, however, differences between field strengths have reached statistical significance. It may highlight the notion that field strengths are a source of variations across studies when VBM is used to investigate age-associated differences in brain structure. 

Note that only age-related effects across field strengths were tested in this study. However, whether similar effects can extend to other comparisons such as sexual dimorphism [10,26] or disease/control [27,28], further studies are a paramount direction for future work. 

Highlighting the limitations of this study that could be improved in future studies is important. First, the scanning parameters used in this cross-field study were not optimized for use at both 3 T and 1.5 T platforms with respect to TR, TE, and FA. Given that the MRI signal intensity depends substantially on the scanning parameters and pulse sequences [8,9], we cannot preclude the possibility that heterogeneous results between field strengths observed in this study could be partially driven by acquisition sequences. Moreover, volume measurements across vendors could also contribute to a volume difference bias [29]. Whether the effects detected in this study can be applied to other platforms requires further investigations. For further exploration, VBM comparisons across scanning protocols and even scanner vendors can be of great interest in future studies. Second, our sample size was small compared with those of other studies [2,3,14,15]. Therefore, the significant clusters did not survive after the multiple comparisons and the interaction between age and field strength was insignificant. However, the less stringent significance threshold of uncorrected *p* < 0.001 can strike a better balance between type I and type II errors [30]. In the case of sample size-dependent age effects, an experiment similar to the experiment in this study but with a larger sample size is suggested. Third, the age-related WM changes are also very important in healthy adults [31], but this phenomenon was not detected in the current study design. This insignificant difference could also be related to our smaller sample size. Fourth, only DARTEL of SPM12 was used in this study. As the type of software/toolbox has significant effects on the VBM results [1,32], possible interactions between VBM algorithms and field strengths should be further explored and investigated.

## 5. Conclusions

In conclusion, we showed that VBM revealed significant age-related changes in the GM volume in a young group at both 3 T and 1.5 T. However, the field strength can have a major influence on VBM results, as the results of age-related changes are heterogeneous between field strengths. Therefore, MRI-instrument-specific factors such as field strength should be considered an important factor when comparing age-specific brain differences across studies.

## Data Availability

The data can be freely given upon request.
